# ﻿Two new species of *Parastagonospora* and a new species of *Phaeoseptoriella* (Phaeosphaeriaceae, Pleosporales) from grasslands in Yunnan Province, China

**DOI:** 10.3897/mycokeys.109.134136

**Published:** 2024-10-10

**Authors:** Ying Gao, Tingfang Zhong, Prapassorn Damrongkool Eungwanichayapant, Ruvishika S. Jayawardena, Kevin D. Hyde, Turki Kh. Faraj, Dhanushka N. Wanasinghe, Heng Gui

**Affiliations:** 1 School of Science, Mae Fah Luang University, Chiang Rai 57100, Thailand; 2 Center of Excellence in Fungal Research, Mae Fah Luang University, Chiang Rai 57100, Thailand; 3 Department of Economic Plants and Biotechnology, Yunnan Key Laboratory for Wild Plant Resources, Kunming Institute of Botany, Chinese Academy of Sciences, Kunming 650201, China; 4 Center for Mountain Futures, Kunming Institute of Botany, Honghe 654400, Yunnan, China; 5 University of Chinese Academy of Sciences, Beijing 100049, China; 6 Department of Botany and Microbiology, College of Science, King Saud University, P.O. Box 22452, Riyadh 11495, Saudi Arabia; 7 Department of Soil Science, College of Food and Agriculture Sciences, King Saud University, P.O. Box 145111, Riyadh 11362, Saudi Arabia

**Keywords:** Ascomycota, coelomycetes, phragmosporous conidia, Poaceae, taxonomy, 3 new species

## Abstract

During our investigation of microfungi on grasslands in Yunnan Province, China, three new fungal taxa associated with grasses were collected. Morphological observations and phylogenetic analyses of the combined SSU, LSU, ITS, *tef1-α*, and *rpb2* loci based on maximum likelihood and Bayesian inference were used to reveal the taxonomic placement of these fungal taxa. This study introduces *Parastagonosporayunnanensis*, *Para.zhaotongensis*, *Phaeoseptoriellapoaceicola. Parastagonosporayunnanensis* is characterized by ampulliform or globose to subglobose conidiogenous cells, with conidia that are cylindrical to subcylindrical, 0–1-septate, rounded at the apex and slightly truncate at the base. *Parastagonosporazhaotongensis* features similar globose to subglobose conidiogenous cells but with 0–3-septate, cylindrical to subcylindrical conidia. *Phaeoseptoriellapoaceicola* is distinguished by its globose to subglobose conidiogenous cells and phragmosporous conidia that are initially hyaline, turn pale yellowish at maturity, and are 7-septate, cylindrical to subcylindrical, either straight or slightly curved. These discoveries underscore the significance of exploring and accurately identifying fungal taxa within Ascomycota, highlighting the species richness and potential for new species discoveries in grass-based habitats. The findings from this study expand our understanding of the taxonomy and phylogeny of grassland-associated Ascomycota, providing a foundation for further ecological and taxonomic studies of these fungi within their natural environments.

## ﻿Introduction

Phaeosphaeriaceae was introduced by Barr (1979) with *Phaeosphaeria* as the type genus and belongs in Dothideomycetes (Wijayawardene et al. 2022). The family is one of the most species-rich families in Dothideomycetes and it includes species that inhabit a wide range of ecosystems, including marine, terrestrial, freshwater, and mangroves (Phookamsak et al. 2014, 2017; Bakhshi et al. 2019; Jones et al. 2019; Luo et al. 2019; Tennakoon et al. 2019). The taxa of Phaeosphaeriaceae are typically endophytic, saprobic, and pathogenic on a wide range of hosts (Barr 1992; Ariyawansa et al. 2013; Phookamsak et al. 2014, 2017, 2019; Hyde et al. 2017; Wanasinghe et al. 2018; Maharachchikumbura et al. 2019; Dissanayake et al. 2022).

*Parastagonospora* was introduced by Quaedvlieg et al. (2013) in Phaeosphaeriaceae (Pleosporales, Dothideomycetes), with *Parastagonosporanodorum* as the type species. There are 27 *Parastagonospora* species listed in Index Fungorum (2024). *Parastagonospora* species are generally identified through their asexual forms. However, only seven species *viz. Para*. *arcana*, *Para.elymi*, *Para.forlicesenica*, *Para.fusiformis*, *Para. jasniorum*, *Para.poaceicola* and *Para. zildae* have been documented in their sexual forms (Li et al. 2016; Thambugala et al. 2017; Goonasekara et al. 2019; Croll et al. 2021).

Species of *Parastagonospora* have been reported from Australia, China, Denmark, Germany, Italy, Iran, the Netherlands, New Zealand, Russia, Turkey, the UK, and the USA as pathogens or saprobes of grasses (Table 1). *Parastagonospora* species are directly and indirectly responsible for significant annual crop losses worldwide. Cunfer (2000) reported that *Parastagonosporaavenae* was a significant pathogen in oats and caused leaf spots in barley and rye. *Parastagonosporanodorum* was reported as a critical pathogen in many countries where wheat and barley are cultivated (Cunfer 2000; Quaedvlieg et al. 2013; Goonasekara et al. 2019; Croll et al. 2021). The list of *Parastagonospora* species reported worldwide is provided in Table 1.

**Table 1.** List of *Parastagonospora* species reported worldwide. NA: data not available.

Species name	Host	Country	Life-Mode	References
*Para.allouniseptata*	*Dactylisglomerata*	Italy	Saprobic	Li et al. (2015)
*Para.Arcana*	*Triticumaestivum*	Iran	Pathogenic	Croll et al. (2021)
*Para.Avenae*	*Loliummultiflorum*, *Avenasativa*	Australia, Germany	Pathogenic	Quaedvlieg et al. (2013); Croll et al. (2021)
*Para.bromicola*	*Bromusinermis*	USA	NA	Croll et al. (2021)
*Para.cumpignensis*	*Dactylisglomerata*	Italy	Saprobic	Li et al. (2016)
*Para.Caricis*	*Phalarisarundinacea*, *Carexacutiformis*Cyperaceae sp.	Netherlands, USA	NA	Quaedvlieg et al. (2013); Fulcher et al. (2018)
*Para.dactylidicola*	*Dactylisglomerata*	Italy	Saprobic	Brahmanage et al. (2020)
*Para.dactylidigena*	*Dactylisglomerata*	Iran	NA	Croll et al. (2021)
*Para.dactylidis*	*Dactylis* sp.	Italy	Saprobic	Li et al. (2015)
*Para.Elymi*	*Elymusrepens*	Russia	Saprobic	Goonasekara et al. (2019)
*Para.forlicesenica*	*Dactylisglomerata*	Italy	Saprobic	Thambugala et al. (2017)
*Para.fusiformis*	*Dactylisglomerata*	Italy	Saprobic	Thambugala et al. (2017)
*Para. golestanensis*	*Agropyrontauri*	Iran	NA	Croll et al. (2021)
*Para.Italica*	*Dactylis* sp.	Italy	Saprobic	Li et al. (2015)
*Para.Jasniorum*	*Triticumaestivum*	Iran	Pathogenic	Croll et al. (2021)
*Para.macrouniseptata*	*Dactylisglomerata*	Italy	Saprobic	Goonasekara et al. (2019)
*Para.Minima*	*Dactylis* sp.	Italy	Saprobic	Li et al. (2015)
*Para.nodorum*	*Loliumperenne*, *Triticumaestivum*, *Leymuschinensis*	Africa, Australia, China, Denmark, North Iran, Turkey, UK, USA	Pathogenic	Quaedvlieg et al. (2013); Zhang and Nan (2018); Croll et al. (2021)
*Para.novozelandica*	Poaceae sp.	New Zealand	NA	Marin-Felix et al. (2019)
*Para.phragmitis*	*Phragmites* sp.	Australia	NA	Marin-Felix et al. (2019)
*Para.poaceicola*	*Dactylisglomerata*	Italy	Saprobic	Thambugala et al. (2017)
*Para.Poae*	*Poa* sp.	Netherlands	NA	Quaedvlieg et al. (2013)
*Para.poagena*	*Poa* sp.	Netherlands	NA	Crous et al. (2014)
*Para.pseudonodorum*	*Triticumaestivum*	Iran	Pathogenic	Croll et al. (2021)
*Para.Stipae*	*Stipapulchra*	USA	NA	Croll et al. (2021)
*Para.uniseptata*	*Daucus* sp.	Italy	Saprobic	Li et al. (2015)
*Para.yunnanensis*	*Loliumperenne*	China	Saprobic	In this study
*Para.zildae*	*Triticumaestivum*	Iran	Pathogenic	Croll et al. (2021)
*Para.zhaotongensis*	*Dactylisglomerata*	China	Saprobic	In this study

*Phaeoseptoriella* was introduced by Crous et al. (2019), with *Phaeoseptoriellazeae* as the type species, on the leaves of *Zeamays* (Poaceae) from South Africa. The asexual morphs of *Phaeoseptoriella* species are characterized by globose, solitary, brown conidiomata with a central ostiole, pale brown, ampulliform to doliiform conidiogenous cells that are smooth with percurrent proliferation at the apex, and pale brown, solitary, fusoid-ellipsoid, straight to slightly curved conidia that are finely roughened with a subobtuse apex, truncate base and are septate (Crous et al. 2019). Tan and Shivas (2023) reported two new species of *Phaeoseptoriella*, *Ph.emmelinepankhurstiae* and *Ph.vidagoldsteiniae*, based on phylogenetic analysis, however, the morphological description was provided. *Phaeoseptoriellaemmelinepankhurstiae* and *Ph.vidagoldsteiniae* were collected from leaves of *Sporobolusnatalensis* (Poaceae) in Australia (Tan and Shivas 2023). *Phaeoseptoriellaedithcowaniae* was collected from a leaf spot of *Heteropogontriticeus* (Poaceae) in Australia (Tan and Shivas 2024).

Grasslands comprise a biome subjected to alternating droughts, where grass and grass-like species dominate (Risser 1988). In the grassland biome, several living organisms, such as insects, herbivorous mammals and fungi (saprobic, pathogenic, and symbiotic), play essential roles in maintaining biodiversity and biomass (Karunarathna et al. 2021). A checklist of Ascomycetes on grasses, which lists 3,165 fungal species, was provided by Karunarathna et al. (2022). Studies of fungi on grasses include those of Thambugala et al. (2017), Goonasekara et al. (2018), and Brahmanage et al. (2020). Some fungi from grass have also been reported in Yunnan province, China. *Dactylellacrassa* was introduced by Miao et al. (1999) from *Oryza* sp. *Hypogymniacongesta* was reported by McCune et al. (2003) from the Poaceae hosts. Yuan et al. (2010) introduced a new species, *Harpophoraoryzae* collected from *Oryzagranulate*. Xia et al. (2013) introduced a novel species *Heteroconiumbannaense* from *Phragmites* sp. *Yunnanensisphragmitis* was reported by Karunarathna et al. (2017). Based on morphology and phylogeny, Gao et al (2022) introduced *Microdochiumgraminearum*, *M.shilinens*, and *M.bolleyi* to the genus *Microdochium*. They were collected from grasses in Yunnan, China. In recent studies, Li et al. (2024) introduced *Anthostomellayunnanensis*, *Astrocystisheterocyclae* and *Collodisculabaoshanensis*, while Dissanayake et al. (2024) introduced *Apiosporaguangdongensis*, *A.locuta-pollinis*, *A.menglaensis*, *A.pseudoparenchymatica*, *Collodisculayunnanensis*, *Digitodochiumailaoshanense* and *D.yunnanense* from bamboo species in Yunnan.

In Yunnan, China, we are continuously surveying the grassland-associated microfungi. Many fungal species may be nearing extinction because they cannot adapt quickly enough to the rapid ecological changes (Wanasinghe et al. 2022; Yasanthika et al. 2023). To mitigate this loss and understand their ecological significance, extensive fungal sampling across various grasslands in different geographic regions is urgently required. Our recent efforts have already yielded several strains of unidentified species isolated from different grass-based hosts (Gao et al. 2022, 2024; Dissanayake et al. 2024), suggesting that there are potentially many new fungal species yet to be discovered in these habitats. Based on morphological illustrations and multi-gene phylogenetic analyses employing ML, and BI, this study introduces three novel species within the Phaeosphaeriaceae. We describe two new species, *Parastagonosporayunnanensis* and *Para.zhaotongensis*, in *Parastagonospora* and a novel species, *Phaeoseptoriellapoaceicola*, to the *Phaeoseptoriella*. The specimens from which these species were collected on *Loliumperenne* and *Dactylisglomerata* were from the grassland areas of Qujing and Zhaotong in Yunnan Province, China.

## ﻿Materials and methods

### ﻿Sample collection, isolation, and morphological observations

Fresh fungal materials were collected from grasslands in Zhaotong and Qujing City, Yunnan Province, China, during the autumn from August to October 2022. The local environment in Zhaotong is characterized by Poaceae as the predominant plant species and features typical plateau vegetation. This area is influenced by a three-dimensional monsoon climate and reaches a maximum elevation of approximately 4000 m (Pei 2022). In contrast, Qujing is characterized by a typical subtropical plateau monsoon climate, with an annual mean temperature of 14.5 °C and average annual precipitation around 1000 mm (Deng et al. 2016). Specimens were stored in plastic Ziplock bags and returned to the mycology laboratory at the Kunming Institute of Botany. Samples were examined using an Olympus SZ-61 dissecting microscope. Fungal fruiting structures were manually sectioned and mounted in water on a slide to observe their microscopic features. Micro-morphological characteristics were examined using a Nikon ECLIPSE Ni-U complex microscope with differential interference contrast (DIC) and phase contrast (PC) illumination. Photos of microscopic structures were captured using a Nikon DS-Ri2 camera. Photo plates and measurements were processed using Adobe Photoshop CS6 Extended version 13.0.1 (Adobe Systems, CA, USA). Single spore isolation of conidia was conducted, and germinated spores were processed by following the methods described in Senanayake et al. (2020). Pure cultures were incubated at 27 °C for two weeks. The living cultures were deposited in the duplicates, which were maintained in the China General Microbiological Culture Collection Center (CGMCC). Herbarium specimens were deposited in the herbarium of the Kunming Institute of Botany Academia Sinica (HKAS). The new taxa have been registered and can be referenced using their Index Fungorum and Faces of Fungi (FoF) numbers as reported by Jayasiri et al. (2015) and in the Index Fungorum (2024). These new taxa are also documented on the Greater Mekong Subregion website (https://gmsmicrofungi.org), as detailed by Chaiwan et al. (2021). Taxonomic novelties were introduced based on a polyphasic approach that integrates morphological, molecular and ecological data, aligning with contemporary taxonomic standards (Chethana et al. 2021; Jayawardena et al. 2021; Maharachchikumbura et al. 2021).

### ﻿DNA extraction, PCR amplification, and sequencing

The extraction of genomic DNA was performed using these fresh mycelia following the methods of Wanasinghe et al. (2016) and Hyde et al. (2023), using the Biospin Fungus Genomic DNA Extraction Kit (BioFlux, Hangzhou, P.R. China) and following manufacturer guidelines. The DNA for the polymerase chain reaction (PCR) was stored at 4 °C for regular use and at -20 °C for long-term usage. Polymerase chain reaction (PCR) was carried out for five genetic markers. The primers and amplification conditions used are listed in Table 2. The total volume of PCR mixtures for amplification was 25 μL containing 8.5 μL ddH2O, 12.5 μL 2 × F8FastLong PCR MasterMix (Beijing Aidlab Biotechnologies Co. Ltd), 2 μL of DNA template, and 1 μL of each forward and reverse primers (stock of 10 pM). The amplified PCR fragments were sent to the Qingke Company, Kunming City, Yunnan Province, China, and Shanghai Sangon Biological Engineering Technology and Service Co., Ltd., China, for sequencing. Sequences were deposited in GenBank.

**Table 2.** Details of genetic markers with PCR primers and thermal cycling program for PCR amplification.

Genetic Marker	Primers	PCR thermal cycle protocols	References
The 18S small subunit rDNA (SSU)	NS1	^a^Annealing at 55 °C for 15 s^c^	White et al. (1990)
	NS4		
The 28S large subunit rDNA (LSU)	LR0R		Rehner and Samuels (1994)
	LR5		Vilgalys and Hester (1990)
The internal transcribed spacers (ITS)	ITS5		White et al. (1990)
	ITS4		
The translation elongation factor 1-alpha (*tef1-α*)	EF1-983F	^a^Annealing at 55 °C for 30 s^c^	Rehner and Buckley (2005)
	EF1-2218R		
The partial RNA polymerase second largest subunit (*rpb2*)	fRPB2-5F	^b^Annealing at 57 °C for 50 s^c^	Liu et al. (1999)
	fRPB2-7cR		

Notes: ^a^ initial denaturation at 94 °C for 3 min, followed by 35 cycles of denaturation at 94 °C for 10 s, elongation at 72 °C for 20 s; ^b^ initial denaturation at 95 °C for 3 min, followed by 35 cycles at 95 °C for 45 s, elongation at 72 °C for 1.5 min; ^c^ final extension at 72 °C for 10 min.

### ﻿Phylogenetic analyses

Sequences obtained from different primers targeting the relevant genes were compared with other sequences sourced from GenBank. A BLAST search identified sequences with high similarity, indicating the closest matches within the Phaeosphaeriaceae taxa and referencing previously published data (Quaedvlieg et al. 2013; Li et al. 2015; Thambugala et al. 2017; Goonasekara et al. 2019; Marin-Felix et al. 2019). The sequences of SSU, LSU, ITS, *tef1-α*, and *rpb2* were downloaded from GenBank (Table 3). Some of the sequences from the study by Croll et al. (2021), which introduced *Parastagonosporabromicola*, *Para. dactylidigena*, *Para. golestanensis*, *Para. jasniorum*, *Para. pseudonodorum* to *Parastagonospora*, were not available in the GenBank. Therefore, we obtained these sequences directly from the first author. The sequences in this study were assembled and manually refined using BioEdit 7.0.9.0 (Hall 1999). The multiple alignments, which included both consensus sequences and reference sequences, were initially generated using MAFFT v. 7 (Kuraku et al. 2013; Katoh et al. 2019). The multiple alignments, which included both consensus sequences and reference sequences, were initially generated using MAFFT v.7. online platform (Katoh et al. 2019) and trimmed with TrimAl v. 1.3 (Capella-Gutiérrez et al. 2009) via the web server Phylemon2 (http://phylemon.bioinfo.cipf.es/utilities.html; accessed on July 10, 2024). and multi-gene alignments were made by the SequenceMatrix program (1.7.8) (Vaidya et al. 2011). Phylogenetic reconstructions of individual and combined datasets were performed using maximum likelihood (ML) and Bayesian inference (BI) analyses on the CIPRES Science Gateway portal (https://www.phylo.org/) (Miller et al. 2012).

**Table 3.** GenBank accession numbers of the strains used for phylogenetic analysis in this study. The new sequences are indicated in bold. Ex-type strains are indicated with the superscript “T”. “NA” is unavailable.

Taxon	Strain numbers	GenBank accession numbers				
		SSU	LSU	ITS	*tef1-α*	*rpb2*
*Dematiopleosporamariae*	MFLU 16-0121	MT226689	MT214576	MT310621	MT394635	NA
*Dematiopleosporamariae*	MFLUCC 13-0612^T^	KJ749652	KJ749653	KX274244	KJ749655	NA
*Dematiopleosporasalsolae*	MFLUCC 17-0828^T^	NG_063679	NG_059184	NR_157514	MG829201	MG829254
*Neosphaerellopsisthailandica*	CPC 21659^T^	NA	NG_067289	NR_137954	NA	NA
*Nodulosphaeriaaconiti*	MFLUCC 13-0728^T^	KU708840	KU708844	NR_154236	KU708852	KU708856
*Nodulosphaeriaguttulatum*	MFLUCC 15-0069	KY501115	KY496726	KY496746	KY514394	KY514405
*Nodulosphaeriascabiosae*	MFLUCC 14-1111^T^	NG_063602	KU708846	NR_154237	KU708854	KU708857
*Paraloratosporamarina*	MFLUCC 19-0691^T^	OQ130107	OQ130110	OQ130046	OQ357219	OQ162221
*Paraloratosporasichuanensis*	KUNCC 23-14218^T^	OR206405	OR206415	OR206396	OR195712	OR195721
*Paraloratosporasichuanensis*	HKAS 129218	OR206406	OR206416	OR206397	OR195713	OR195722
*Parastagonosporaallouniseptata*	MFLUCC 13-0386^T^	NA	KU058721	KU058711	MG520914	NA
*Parastagonosporaavenae*	CBS 289.69	NA	KF251678	KF251174	NA	KF252182
*Parastagonosporaavenae*	CBS 290.69	NA	KF251679	KF251175	NA	KF252183
*Parastagonosporacaricis*	CBS 135671^T^	NA	KF251680	KF251176	NA	KF252184
*Parastagonosporadactylidicola*	MFLU 20-0387^T^	NA	MT370430	MT370412	NA	NA
*Parastagonosporadactylidis*	MFLUCC 13-0375^T^	NA	KU058722	KU058712	NA	NA
*Parastagonosporadactylidis*	MFLUCC 13-0376	MG520986	KU058723	KU058713	MG520916	NA
*Parastagonosporadactylidis*	MFLUCC 13-0573	KU842390	KU842389	KU842388	NA	NA
*Parastagonosporaelymi*	KUMCC 16-0125^T^	NA	MN002870	MN002867	NA	NA
*Parastagonosporaforlicesenica*	MFLUCC 13-0557^T^	NA	KY769661	KY769660	NA	NA
*Parastagonosporafusiformis*	MFLUCC 13-0215^T^	NG_068367	NG_068235	NR_165848	NA	KX863711
*Parastagonosporaitalica*	MFLUCC 13-0377^T^	MG520985	KU058724	KU058714	MG520915	NA
*Parastagonosporamacrouniseptata*	KUMCC 16-0111^T^	NA	MN002868	MN002869	NA	MN019669
*Parastagonosporanodorum*	CBS 110109	EU754076	KF251681	KF251177	NA	KF252185
*Parastagonosporanovozelandica*	CPC 29613^T^	NA	MK540028	MK539957	NA	MK540088
*Parastagonosporaphragmitis*	CPC 32075^T^	NA	NG_066451	NR_164454	NA	MK540089
*Parastagonosporapoaceicola*	MFLUCC 15-0471^T^	NG_068368	NG_068537	NA	NA	KX880499
*Parastagonosporapoae*	CBS 135091	NA	KF251683	KF251179	NA	KF252187
*Parastagonosporapoae*	CBS 135089^T^	NA	KF251682	KF251178	NA	KF252186
*Parastagonosporapoagena*	CBS 136776^T^	NA	KJ869174	KJ869116	NA	NA
*Parastagonosporastipae*	pn1617	NA	NA	MW263184	NA	NA
*Parastagonosporauniseptata*	MFLUCC 13-0387^T^	MG520987	KU058725	KU058715	MG520917	NA
***Parastagonosporayunnanensis***	**CGMCC 3.24527^T^**	**PQ046289**	**PQ046315**	**PQ046302**	**PQ058300**	**PQ058313**
***Parastagonosporayunnanensis***	**CGMCC 3.24528**	**PQ046290**	**PQ046316**	**PQ046303**	**PQ058301**	**PQ058314**
***Parastagonosporayunnanensis***	**CGMCC 3.24529**	**PQ046291**	**PQ046317**	**PQ046304**	**PQ058302**	**PQ058315**
***Parastagonosporayunnanensis***	**CGMCC 3.24530**	**PQ046292**	**PQ046318**	**PQ046305**	**PQ058303**	**PQ058316**
***Parastagonosporayunnanensis***	**CGMCC 3.24511**	**PQ046285**	**PQ046311**	**PQ046298**	**PQ058296**	**PQ058309**
***Parastagonosporayunnanensis***	**CGMCC 3.24512**	**PQ046286**	**PQ046312**	**PQ046299**	**PQ058297**	**PQ058310**
***Parastagonosporazhaotongensis***	**CGMCC 3.24519^T^**	**PQ046287**	**PQ046313**	**PQ046300**	**PQ058298**	**PQ058311**
***Parastagonosporazhaotongensis***	**CGMCC 3.24520**	**PQ046288**	**PQ046314**	**PQ046301**	**PQ058299**	**PQ058312**
*Phaeoseptoriellaedithcowaniae*	BRIP 75864a^T^	NA	PP708933	PP707905	NA	NA
*Phaeoseptoriellaemmelinepankhurstiae*	BRIP65639a^T^	NA	NA	OR673891	NA	NA
***Phaeoseptoriellapoaceicola***	**CGMCC 3.24561^T^**	**PQ046283**	**PQ046309**	**PQ046296**	**PQ058294**	**PQ058307**
***Phaeoseptoriellapoaceicola***	**CGMCC 3.24562**	**PQ046284**	**PQ046310**	**PQ046297**	**PQ058295**	**PQ058308**
***Phaeoseptoriellapoaceicola***	**CGMCC 3.25058**	**PQ046293**	**PQ046319**	**PQ046306**	**PQ058304**	**PQ058317**
***Phaeoseptoriellapoaceicola***	**CGMCC 3.25059**	**PQ046294**	**PQ046320**	**PQ046307**	**PQ058305**	**PQ058318**
***Phaeoseptoriellapoaceicola***	**CGMCC 3.25060**	**PQ046295**	**PQ046321**	**PQ046308**	**PQ058306**	**PQ058319**
*Phaeoseptoriellavidagoldsteiniae*	BRIP65641a^T^	NA	NA	OR673892	NA	NA
*Phaeoseptoriellazeae*	CBS 144614^T^	NA	NG_067869	NR_163371	NA	MK442674
*Phaeosphaeriachengduensis*	KUNCC 23-13571^T^	OR206401	OR206411	OR206392	OR195708	OR195717
*Phaeosphaeriachiangraina*	MFLUCC 13-0231^T^	KM434289	NG_069237	NR_155643	KM434298	KM434307
*Phaeosphaeriasichuanensis*	KUNCC 23-13569^T^	OR206399	OR206409	OR206390	OR195706	OR195715
*Phaeosphaeriathysanolaenicola*	MFLUCC 10-0563^T^	KM434286	NG_069236	NR_155642	KM434295	KM434303
*Quixadomyceshongheensis*	KUMCC 20-0215^T^	NG_074964	MW264194	NR_172441	MW256816	MW269529
*Quixadomyceshongheensis*	HKAS 112346	MW541833	MW541822	MW541826	MW556134	MW556136
*Sclerostagonosporalathyri*	MFLUCC 14-0958^T^	NG_063692	NG_069566	NR_158956	MG829235	NA
*Sclerostagonosporarosicola*	MFLUCC 15-0129^T^	NG_063693	MG829068	MG828957	MG829237	NA
*Septoriellaarundinicola*	MFLU 16-0225^T^	NG_062199	MG829056	MG828946	MG829228	MG829261
*Septoriellaasparagicola*	MFLUCC 16-0379^T^	NG_067708	NG_070081	NR_165908	MK443385	MK443387
*Septoriellaneodactylidis*	MFLUCC 14-0966^T^	NG_061288	NG_069554	NR_157511	MG829199	MG829253
*Wojnowiciellaclematidis*	MFLUCC 17-2159^T^	MT226695	MT214582	NR_170812	MT394641	MT394698
*Wojnowiciellakunmingensis*	KUMCC 18-0159^T^	NG_067701	NG_070079	NR_164446	MK359071	MK359078

Maximum likelihood trees were inferred using RAxML-HPC2 on XSEDE v. 8.2.12 (Stamatakis 2014) and used the GTR+GAMMA model of nucleotide evolution with 1000 bootstrap replicates. Bayesian inference analysis was conducted using MrBayes on XSEDE (3.2.7a) (Ronquist et al. 2012). The alignments containing SSU, LSU, ITS, *tef1-α*, and *rpb2* were converted to NEXUS format (.nxs) using CLUSTAL X (2.0) and PAUP v. 4.0b10 (Thompson et al. 1997; Swofford 2002). The evolutionary models for BI analysis were selected independently for each locus using MrModeltest v. 2.3 (Nylander et al. 2008) under the Akaike information criterion as follows: GTR+I+G substitution model was chosen for ITS, LSU, *tef1-α*, and *rpb2*, HKY substitution model was selected for SSU. Markov Chain Monte Carlo sampling (MCMC) was used to determine posterior probabilities (PP) (Zhaxybayeva and Gogarten 2002). Six simultaneous Markov chains were run for five million generations, and trees were sampled every 200^th^ generation. The first 25% of trees were considered burn-in and discarded. The two runs were considered convergent when the standard deviation of split frequencies dropped below 0.01. The Fig. Tree version 1.4.0 program (Rambaut and Drummond 2012) was used to visualize the phylogenetic trees and reorganized them in Microsoft PowerPoint before being saved in PDF format and, finally, converted to TIFF format using Adobe Photoshop CS6 Extended version 13.0.1 (Adobe Systems, CA, USA).

## ﻿Results

### ﻿Phylogenetic analysis

**Figure 1.**
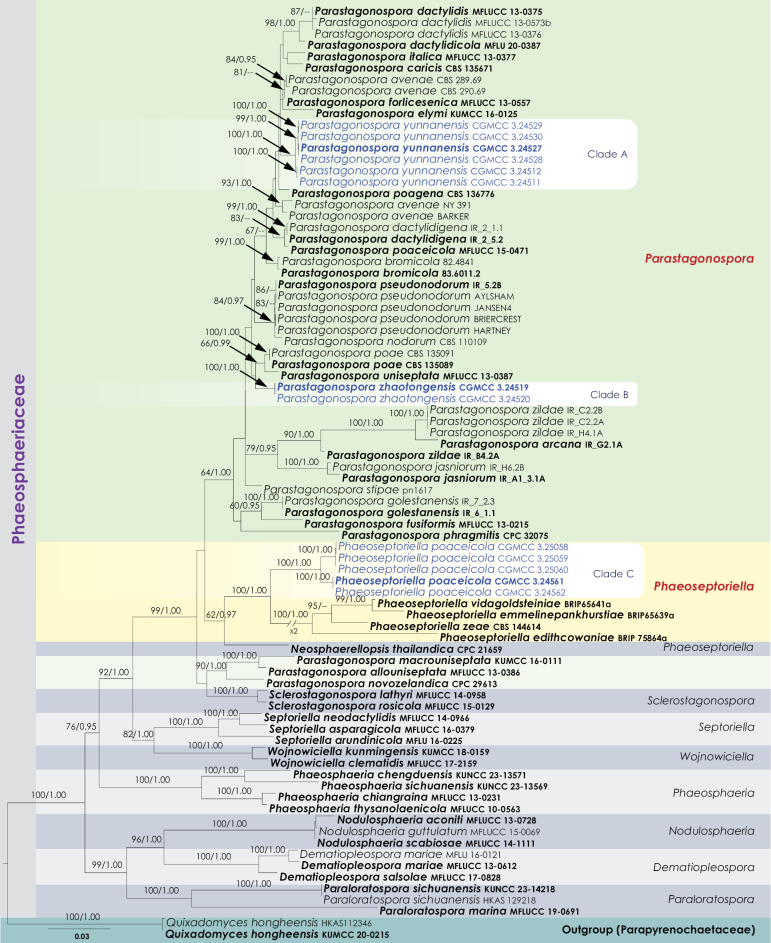
Phylogenetic tree obtained from combined SSU, LSU, ITS, *tef1-α*, and *rpb2* sequence data. The tree is rooted with *Quixadomyceshongheensis* (HKAS 112346 and KUMCC 20-0215). Bootstrap support values for ML equal to or greater than 60% and the Bayesian posterior probabilities equal to or higher than 0.95 PP are indicated above the nodes as ML/PP. Ex-type strains are indicated in bold, and the new isolates are highlighted in blue.

The combined sequence data of SSU, LSU, ITS, *tef1-α*, and *rpb2*, comprised 82 strains including the outgroup (Fig. 1). A total of 4,292 characters, including gaps, were obtained in the phylogenetic analysis *viz.* SSU = 1–965 bp, LSU = 966–1,756 bp, ITS = 1,757–2,378 bp, *tef1-α* = 2,379–3,160 bp, *rpb2* = 3,161–4,292 bp. The RAxML analysis of the combined dataset yielded a best-scoring tree with a final ML optimization likelihood value of -25260.798404. The matrix had 1,382 distinct alignment patterns, with 31.47% undetermined characters or gaps. The estimated base frequencies were as follows: A = 0.245359, C = 0.244397, G = 0.264796, T = 0.245448; substitution rates: AC = 1.259544, AG = 3.943431, AT = 1.705789, CG = 0.844343, CT = 7.018866, GT = 1.000000, proportion of invariable sites I = 0.644641; and gamma distribution shape parameter α = 0.619642.

The Bayesian analysis proceeded for 783,000 generations, achieving an average standard deviation for split frequencies below 0.01 (0.009957). This analysis produced a total of 7,831 trees. After discarding the first 25% as burn-in, 5,874 trees were sampled for further consideration. The alignment included 1,383 unique site patterns. Both BI and ML trees were consistent with each other; the ML tree is presented in Fig. 1. Where relevant, the phylogenetic findings shown in Fig. 1 are further discussed in the descriptive notes that follow.

Except for *Parastagonosporaallouniseptata* (MFLUCC 13-0386), *Para.macrouniseptata* (KUMCC 16-0111) and *Para.novozelandica* (CPC 29613), all other *Parastagonospora* strains nested within a monophyletic clade supported by 64% ML and 1.00 BYPP. Within this clade, the new strains CGMCC 3.24511, CGMCC 3.24512, CGMCC 3.24527, CGMCC 3.24528, CGMCC 3.24529, and CGMCC 3.24530 formed a distinct monophyletic clade, achieving 100% ML and 1.00 BYPP bootstrap support (Clade A, Fig. 1). Additionally, CGMCC 3.24519 and CGMCC 3.24520 grouped together with 100% ML and 1.00 BYPP bootstrap support (Clade B, Fig. 1), positioned as sister to *Parastagonosporapoae* (CBS 135089, CBS 135091) and *Para.uniseptata* (MFLUCC 13-0387). However, this sister relationship was not statistically supported (Fig. 1).

*Phaeoseptoriellaedithcowaniae* (BRIP 75864a), *Ph.emmelinepankhurstiae* (BRIP65639a), *Ph.vidagoldsteiniae* (BRIP65641a), *Ph.zeae* (CBS 144614) grouped with our new isolates, CGMCC 3.24561, CGMCC 3.24562, CGMCC 3.25058, CGMCC 3.25059 and CGMCC 3.25060. All of these new isolates clustered in a distinct monophyletic clade, achieving 100% ML and 1.00 BYPP bootstrap support (Clade C, Fig. 1).

### ﻿Taxonomy

#### ﻿ Parastagonospora yunnanensis

Taxon classificationFungiPleosporalesPhaeosphaeriaceae

Y. Gao, H. Gui & K.D. Hyde sp. nov.

249C7016-9A4F-5429-8E7E-98B6C065A69C

Index Fungorum: IF902468

Facesoffungi Number: FoF16258

Fig. 2

##### Etymology.

The specific epithet “yunnanensis” refers to Yunnan Province, where the holotype was collected.

##### Holotype.

HKAS 128771.

##### Description.

Saprobic on decaying stem of *Loliumperenne* (Poaceae). ***Sexual morph***: Undetermined. ***Asexual morph*: *Conidiomata*** 35–45 µm high, 120–140 μm diam. (x– = 40.6 × 132.7 μm, n = 10), solitary, flattened, subglobose to irregular oval, brown to dark brown spots, immersed in the epidermis of the host, ostiolate. ***Conidiomata wall*** 4–13 µm wide (x– = 9 μm, n = 25), composed of brown cells of textura angularis, with an inner layer comprising hyaline cells. ***Conidiogenous cells*** (3.2–)3.5–4.7(–5.3) × (3.4–)4–5.3(–6.1) μm (x– = 4 ± 0.57 × 4.77 ± 0.59 μm, n = 30), hyaline, ampulliform or globose to subglobose, smooth-walled. ***Conidia*** (16.2–)18–20(–20.4) × (3–)3.2–3.7(–4) μm (x– = 19 ± 1.1 × 3.4 ± 0.24 μm, n = 35), hyaline, 0–1-septate, cylindrical to subcylindrical, rounded at apex, slightly truncate at base, guttulate, smooth-walled.

##### Culture characteristics.

*Conidia* germinated on PDA within 24 hours, and a germ tube was initially produced from the ends of the conidia. *Colonies on PDA* reaching 20 mm in 3 weeks at room temperature (25–27 °C), regular, floccose, white from the above and light grey from the centre and below, smooth with a filamentous edge.

**Figure 2.**
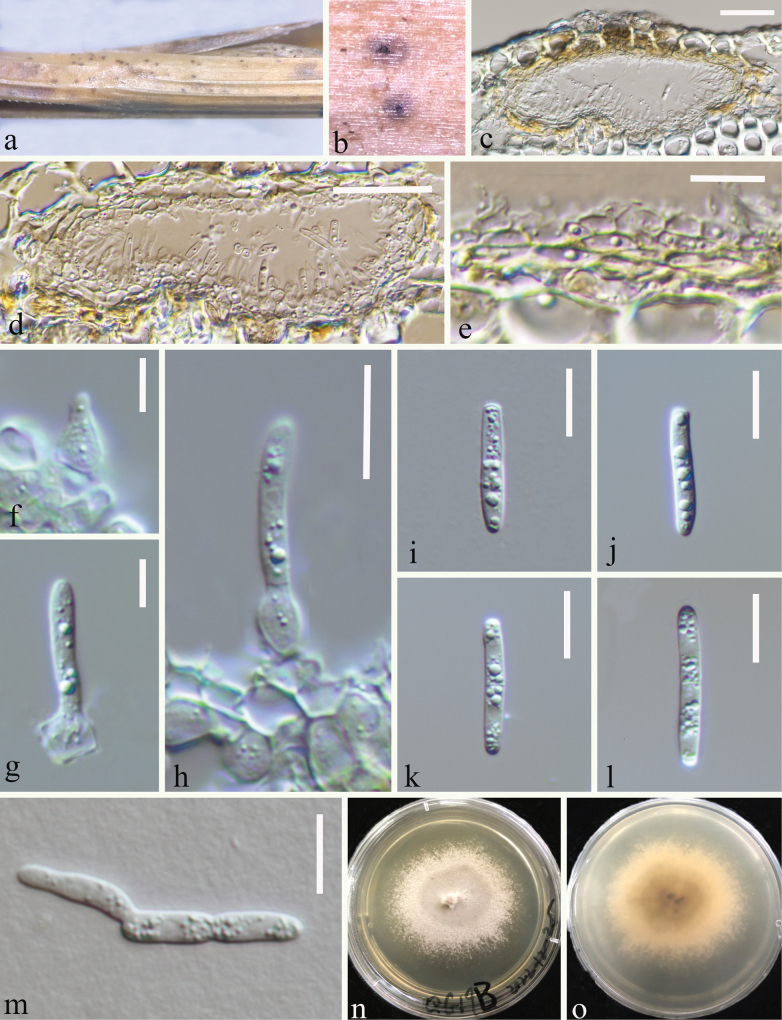
Asexual morph of *Parastagonosporayunnanensis* (HKAS 128771, holotype) on a dead stalk of *Loliumperenne***a, b** conidiomata on the host **c, d** vertical section of conidiomata **e** conidioma wall **f–h** conidiogenous cells arise from the wall and develop conidia **i–l** conidia **m** germinating conidium **n** cultures on PDA from above **o** cultures on PDA from the reverse. Scale bars: 30 μm (**c, d**); 10 μm (**e, h–m**); 5 μm (**f, g**).

##### Material examined.

China • Yunnan Province, Zhaotong City, (26°56'39"N, 103°8'53"E), on a decaying stem of *Loliumperenne* (Poaceae), 25 August 2022, Ying Gao, QG69A (HKAS 128771, holotype), ex-type (CGMCC 3.24527) • *ibid.* QG69B (HKAS 128772, paratype), ex-paratype (CGMCC 3.24528) • *ibid*. QG71A (HKAS 128773), culture (CGMCC 3.24529) • *ibid*. QG71B (HKAS 128774), living culture (CGMCC 3.24530); *ibid.* • Qujing City, (26°21'31"N, 103°14'13"E), on a decaying stem of *Loliumperenne* (Poaceae), 27 August 2022, Ying Gao, QG19A (HKAS 128799), living culture (CGMCC 3.24511) • *ibid*. QG19B (HKAS 128800), living culture (CGMCC 3.24512).

##### Notes.

*Parastagonosporayunnanensis* is introduced as a new species based on its distinct morphology and phylogenetic analysis of combined SSU, LSU, ITS, *tef1-α*, and *rpb2* datasets. We have collected six isolates of this fungus from both the Qujing and Zhaotong regions. *Parastagonosporayunnanensis* is phylogenetically related to *Parastagonosporaelymi* (KUMCC 16-0125). *Parastagonosporaelymi* was introduced as a saprobic fungus from *Elymusrepens* in Russia, the asexual morph of *Parastagonosporaelymi* has not been determined (Goonasekara et al. 2019). In addition, the ITS pairwise nucleotide comparison of these species showed 18/497 bp differences (3.62%, with 2 gaps), the comparison of base pairs in LSU showed 0.36% differences (3/834 bp, without gaps), SSU, *tef1-α*, *and rpb2* of *Parastagonosporaelymi* were not provided.

#### ﻿ Parastagonospora zhaotongensis

Taxon classificationFungiPleosporalesPhaeosphaeriaceae

Y. Gao, H. Gui & K.D. Hyde sp. nov.

C80654EA-E062-5F6A-874C-F9742BCB1737

Index Fungorum: IF902469

Facesoffungi Number: FoF16259

Fig. 3

##### Etymology.

The specific epithet “zhaotongensis” refers to Zhaotong City, where the holotype was collected.

##### Holotype.

HKAS 132983.

##### Description.

Saprobic on decaying stem of *Dactylisglomerata* (Poaceae). ***Sexual morph***: Undetermined. ***Asexual morph*: *Conidiomata*** 70–85 μm high × 80–110 μm diam (x– = 76 × 94 μm, n = 10) 80–110 μm diam × 70–85 μm high (x– = 94 × 76 μm, n = 10), flattened, solitary, immersed in the epidermis of the host, globose to subglobose, brown to dark brown spots. ***Conidiomatal wall*** 5–13 µm wide (x– = 8 μm, n = 30), thin wall, 2–3 layered, composed of pale brown cells of textura angularis, with inner layer comprising hyaline cells. ***Conidiogenous cells*** (3.5–)4.5–6(–6.5) × (3–)4–5.5(–6) μm (x– = 5.5 ± 0.74 × 5 ± 0.77 μm, n = 20), hyaline, globose to subglobose, smooth-walled. ***Conidia*** (22–)25–30(–32)× (2.7–)3–3.4(–3.7) μm (x– = 28 ± 2.45 × 3.3 ± 0.21 μm, n = 35), hyaline, 0–3-septate, cylindrical to subcylindrical, smooth-walled, rounded at apex, slightly truncate at base, guttulate.

**Figure 3.**
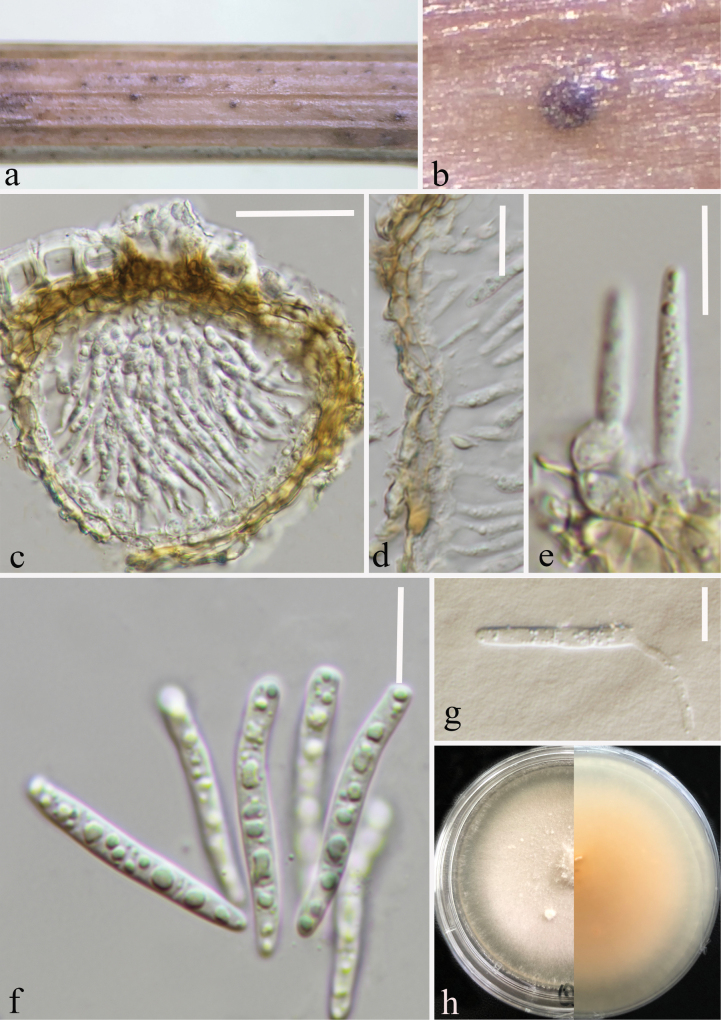
Asexual morph of *Parastagonosporazhaotongensis* (HKAS 132983, holotype) on a dead stalk of *Dactylisglomerata***a, b** conidiomata on the host **c** vertical section of conidioma **d** conidioma wall **e** conidiogenous cells arise from the wall and develop conidia **f** conidia **g** germinating conidium **h** cultures on PDA from above and reverse. Scale bars: 30 μm (**c**); 15 μm (**d**); 10 μm (**e–g**).

##### Culture characteristics.

Colonies on PDA, reaching 20–25 mm diam., after three weeks at 25–27 °C, with circular, floccose, white from the above and in reverse pale yellow.

##### Material examined.

China • Yunnan Province, Qujing City (26°37'38"N, 103°15'29"E), on decaying stem of *Dactylisglomerata* (Poaceae), 27 August 2022, Ying Gao, QG44A (HKAS 132983, holotype), ex-type (CGMCC 3.24519) • *ibid.* QG44B (HKAS 132984, paratype), ex-paratype (CGMCC 3.24520).

##### Note.

Based on multi-locus phylogenetic analyses, our strains of *Parastagonosporazhaotongensis* (CGMCC 3.24519 and CGMCC 3.24520) are closely related to *Para.uniseptata* (MFLUCC 13-0387) and *Para.poae*. *Parastagonosporauniseptata* was reported on *Daucus* sp. from Italy by Li et al. (2015). Pairwise nucleotide comparison indicates that our strains differ from *Parastagonosporauniseptata* in 17/573 bp of ITS (2.97%, with 4 gaps), 2/834 bp of LSU (0.24%, without gaps) and 20/906 bp of *tef*1-α (2.21%, without gaps). The *rpb*2 sequence of *Parastagonosporauniseptata* was not available for comparisons. Morphologically, *Parastagonosporazhaotongensis* is distinguished by its conidiogenous cells (globose to subglobose vs. ampulliform to broadly conical, phialidic), and conidia (22–32 μm long, 0–3-septate vs. 14–18 μm long, 1-septate) (Table 4). The pairwise nucleotide comparison showed that our strains (CGMCC 3.24519 and CGMCC 3.24520) differ from *Parastagonosporapoae* (CBS 135089) in 15/562 bp of ITS (2.67%, with 4 gaps), 2/828 bp of LSU (0.24%, without gaps), and 10/250 bp of *rpb*2 (4.00%, without gaps). SSU and *tef*1-α data for *Parastagonosporapoae* were not provided. *Parastagonosporazhaotongensis* differs from *Para.poae* in conidiomata (80–110 μm in diam., brown to dark brown spots *vs.* up to 250 μm in diam. black), conidiogenous cells (3.5–6.5 μm long, globose to subglobose *vs.* 6–10 μm long, ampulliform to subcylindrical) and conidia (2.7–3.7 μm wide, 0–3-septate *vs.* 2–2.5 μm wide, 1-septate) (Table 4).

**Table 4.** Synopsis of asexual morphological characters of *Parastagonospora* species.

Name of Taxon	Conidiomata size (μm)	Conidiogenous cells		Conidia			Reference
		Shape	Size (μm)	Shape	Size (μm)	septa	
*Para.allouniseptata*	60–90 × 70–90	Ampulliform, phialidic	3–5 × 3–5.5	Subcylindrical, subobtuse apex, truncate base	16–22 × 2.5–3.5	1	Li et al. (2015)
*Para.avenae*	60–90	Ampulliform	7–10 × 3–5	Subcylindrical, truncate base with obtuse apex	4–6 × 2	0	Croll et al. (2021)
*Para.bromicola*	150–200	Ampulliform to subcylindrical	4–6 × 4–5	Subcylindrical, subobtuse apex, truncate base	12–18 × 2–3	1(–3)	Croll et al. (2021)
*Para.caricis*	Up to 250	Ampulliform, phialidic	8–15 × 4–6	Subcylindrical, subobtuse apex, truncate base, scolecosporous	50–75 × 5–6	7–15	Quaedvlieg et al. (2013)
*Para.dactylidicola*	100–110 × 85–115	Ampulliform to subcylindrical, broadly cylindrical or conical, phialidic	–	Hyaline or subhyaline, ellipsoid to oblong, or subcylindrical, with obtuse or subobtuse apex	7.5–10 × 2.5–3.5	1	Brahmanage et al. (2020)
*Para.dactylidigena*	250–350	Ampulliform to subcylindrical	5–7 × 4–5	Subcylindrical, subobtuse apex, truncate base	25–42 × 4–5	3(–6)	Croll et al. (2021)
*Para.dactylidis*	50–100 × 100–150	Ampulliform, phialidic	2–6 × 3–8	Fusiform, curved, rounded at both ends	25–40 × 4–5.5	3	Li et al. (2015)
*Para.golestanensis*	200–350	Ampulliform	5–10 × 4–5	Subcylindrical, subobtuse apex, truncate base	22–35 × 2.5–3	(1–)3	Croll et al. (2021)
*Para.italica*	65–80 × 40–150	Broadly cylindrical, phialidic	–	Cylindric-fusiform, with narrow and obtuse apex, truncate base	25–32 × 3–4	3-euseptate	Li et al. (2015)
*Para.jasniorum*	250–300	Ampulliform, phialidic, aggregated	5–6 × 4–5	Subcylindrical, apical cell with slight taper to subobtuse apex	22–35 × 2.5–3	(1–)3(–5)	Croll et al. (2021)
*Para.macrouniseptata*	120–160 × 150–190	Ampulliform to lageniform, phialidic, discrete	4.2 × 3	Cylindrical to subcylindrical, rounded at apex, truncate base	14–20 × 1–2.5	1	Goonasekara et al. (2019)
*Para.minima*	40–70 × 50–100	Ampulliform, phialidic	3–6.5 × 3–7	Subcylindrical, slightly curved, wider at the basal half, narrow, and rounded at both ends	20–28 × 3.5–4.5	3-euseptate	Li et al. (2015)
*Para.nodorum*	10–15	Globose to ampulliform	5–7 × 4–6	Subcylindrical, subobtuse apex, truncate base	11–28 × 2.5–4	1–3	Croll et al. (2021)
*Para.novozelandica*	180–200	Ampulliform to subcylindrical	6–8 × 2.5–5	Subobtuse apex, truncate base, subcylindrical	9–16 × 2–3	1	Marin-Felix et al. (2019)
*Para.phragmitis*	250–300	Ampulliform to doliiform	7–10 × 8–9	Hyaline to pale olivaceous, subcylindrical-fusoid	18–27 × 3–4	3	Marin-Felix et al. (2019)
*Para.poae*	up to 250	Ampulliform to subcylindrical, phialidic, aggregated	6–10 × 3–5	Truncate base, cylindrical, thin-walled, with obtuse apex	20–32 × 2–2.5	1	Quaedvlieg et al. (2013)
*Para.poagena*	up to 350	Ampulliform to subcylindrical	4–6 × 3–6	Subcylindrical, truncate base, sigmoid	30–60 × 3–4	3–9	Crous et al. (2014)
*Para.pseudonodorum*	200–350	Ampulliform to subcylindrical	4–9 × 4–6	Cylindrical, subobtuse apex	27–36 × 2.5–4	3	Croll et al. (2021)
*Para.stipae*	150–180	Ampulliform to subcylindrical	5–6 × 3–4	Subcylindrical, subobtuse apex	8–18 × 2.5–3	1	Croll et al. (2021)
*Para.uniseptata*	60–100 × 70–100	Ampulliform to broadly conical, phialidic	3–6 × 3–6.5	Subcylindrical, truncate base with obtuse apex	14–18 × 2–3	1	Li et al. (2015)
*Para.yunnanensis*	120–140 × 35–45	Ampulliform or globose to subglobose	3.2–5.3 × 3.4–6.1	Cylindrical to subcylindrical	16–20 × 3–4	0–1	In this study
*Para.zhaotongensis*	80–110 × 70–85	Globose to subglobose	3.5–6.5 × 3–6	Subcylindrical, rounded at apex, truncate base	22–32 × 3–4	0–3	In this study

#### ﻿ Phaeoseptoriella poaceicola

Taxon classificationFungiPleosporalesPhaeosphaeriaceae

Y. Gao, H. Gui & K.D. Hyde sp. nov.

247AD12B-F2A1-5475-8FDA-35DFA8C9CD47

Index Fungorum: IF902470

Facesoffungi Number: FoF16260

Fig. 4

##### Etymology.

in reference to the holotype occurring on grasses (Poaceae)

##### Holotype.

HKAS 128741.

##### Description.

Saprobic on decaying stem of *Dactylisglomerata* (Poaceae). ***Sexual morph***: Undetermined. ***Asexual morph*: *Conidiomata*** 60–75 μm high × 90–100 μm diam. (x– = 70 × 97 μm, n = 15), flattened, solitary, immersed in the epidermis of the host, globose to subglobose, brown to black spots. ***Conidiomatal wall*** 5.5–13 µm wide (x– = 9.5 μm, n = 25), thin wall, 1–4 layered, composed of pale brown cells of textura angularis, with inner layer comprising hyaline cells. ***Conidiogenous cells*** (4.1–)4–7.7(–9.1) × (4–)4.3–5.7(5.5) μm (x– = 5.9 ± 1.82 × 5 ± 0.70 μm, n = 25), hyaline, globose to subglobose, smooth-walled. ***Conidia*** (30–)33–39(–41) × (4.3–)5–6(–6.7) μm (x– = 36 ± 3.23 × 5.5 ± 0.56 μm, n = 35), phragmosporous, initially hyaline, becoming pale yellowish at maturity, 7-septate, cylindrical to subcylindrical, straight or slightly curved, smooth-walled, rounded at apex, slightly truncate at base, guttulate.

**Figure 4.**
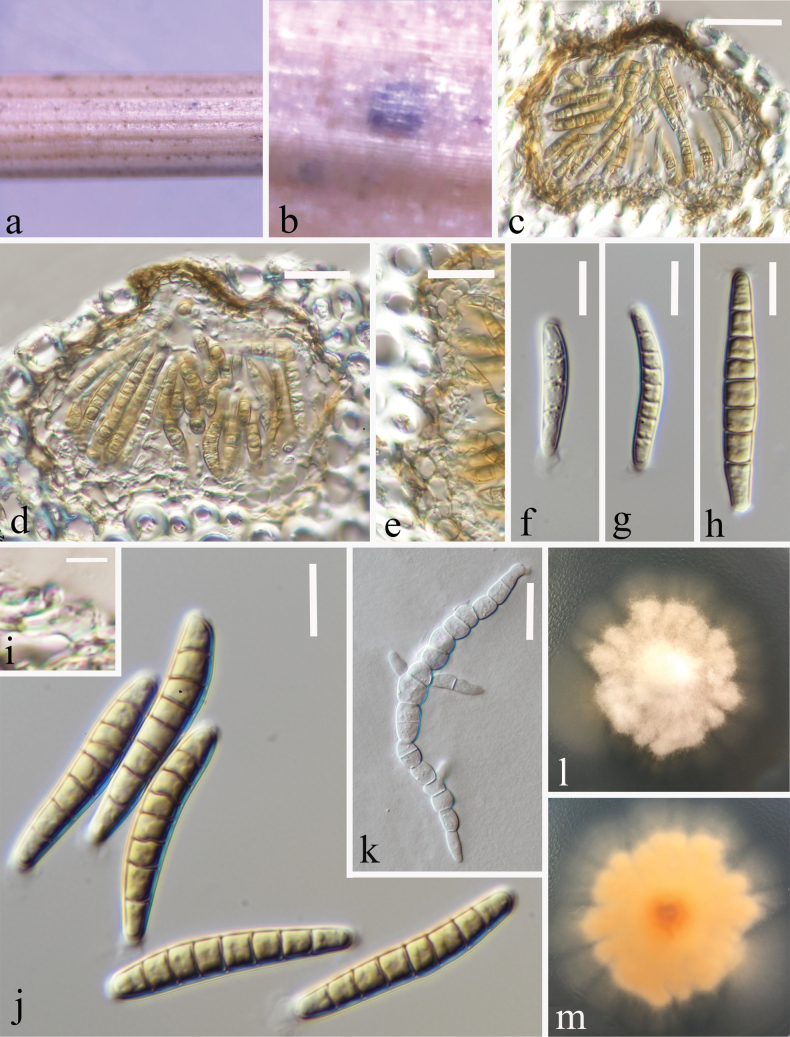
Asexual morph of *Phaeoseptoriellapoaceicola* (HKAS 128741, holotype) on a dead stalk of *Dactylisglomerata***a, b** conidiomata on the host **c, d** vertical section of conidiomata **e** conidioma wall **i** conidiogenous cell arise from the wall and develop conidium **f–h, j** conidia **k** germinating conidium **l** cultures on PDA from above **m** cultures on PDA from the reverse. Scale bars: 30 μm (**c**); 20 μm (**d, e, k**); 10 μm (**f–h, j**); 5 μm (**i**).

##### Culture characteristics.

Colonies on PDA, reaching 10–20 mm diam., after three weeks at 25–27 °C, with irregular, floccose, raised, white from the above and in reverse yellow.

##### Material examined.

China • Yunnan Province, Zhaotong City (27°38'37"N, 103°37'5"E), on decaying stem of *Dactylisglomerata* (Poaceae), 25 September 2022, Ying Gao, LG7A (HKAS 128741, holotype), ex-type (CGMCC 3.24561) • *ibid.* LG7B (HKAS 128742, paratype), ex-paratype (CGMCC 3.24562). *ibid.* • China, Yunnan Province, Zhaotong City, (27°26'34"N,103°19'16"E), on decaying stems of *Anaphalistenuisissima*, 20 August 2021, Ying Gao, living cultures: ZY356B (CGMCC 3.25058), ZY359A (CGMCC 3.25059), ZY359B (CGMCC 3.25060).

##### Note.

*Phaeoseptoriellapoaceicola* is introduced as a new species based on morphology and phylogenetic analysis of combined SSU, LSU, ITS, *tef1-α*, and *rpb2* datasets. Our strains of *Phaeoseptoriellapoaceicola* (CGMCC 3.24561, CGMCC 3.24561, CGMCC 3.25058, CGMCC 3.25059, and CGMCC 3.25060) distinct clade (100% ML, 1.00 PP, Clade C, Fig. 1). The ITS pairwise nucleotide comparison of our isolate *Phaeoseptoriellapoaceicola* (CGMCC 3.24561) with *Phaeoseptoriellaedithcowaniae* (PP707905), *Ph.emmelinepankhurstiae* (OR673891), *Ph.vidagoldsteiniae* (OR673892), *Ph.zeae* (NR_163371) showed 123/601 bp, 83/537 bp, 81/537 bp, 88/534 bp, differences (20.47%, with 37 gaps; 15.46%, with 27 gaps; 15.08%, with 25 gaps; 16.48%, with 28 gaps), respectively. Our isolate differs from *Ph.edithcowaniae* (PP708933) and *Ph.zeae* (NG_067869) in 13/836 bp and 4/836 (1.56% and 0.48% without gaps) in the LSU regions, respectively. It differs from *Ph.zeae* (MK442674) in 130/878 bp (14.81%, without gaps) in the *rpb2* regions, SSU and *tef1-α* of other *Parastagonospora* species were not provided. *Phaeoseptoriella* only included four species, however, *Ph.emmelinepankhurstiae*, *Ph.edithcowaniae*, and *Ph.eidagoldsteiniae* have not provided with morphological analyses (Tan and Shivas 2023, 2024). *Phaeoseptoriellapoaceicola* differs from *Ph.zeae* in comparatively smaller conidiomata (90–100 μm diam. vs. 200–250 μm diam.), conidiogenous cells (hyaline, globose to subglobose vs. pale brown, ampulliform to doliiform), bigger conidia (30–41 μm long, 4–7 μm wide, cylindrical to subcylindrical, 7-septate, vs. 14–23 μm long, 3–4 μm wide, fusoid-ellipsoid, 3-septate) (Crous et al. 2019). Based on the guidelines for a polyphasic approach recommended for species boundary delimitation (Chethana et al. 2021; Maharachchikumbura et al. 2021), we introduce *Phaeoseptoriellapoaceicola* as a novel taxon.

## ﻿Discussion

This study refines the taxonomic classification of microfungi in grasslands across Yunnan Province, southwestern China, by identifying and characterizing three new fungal species *viz. Parastagonosporayunnanensis*, *Para.zhaotongensis*, and *Phaeoseptoriellapoaceicola*. Our taxonomic approach incorporates a multi-locus sequence analysis utilizing five gene loci (SSU, LSU, ITS, *tef*1-α, and *rpb*2) crucial for discerning species boundaries in genera where morphological characteristics are either overlapping or inadequate for clear species differentiation (Wanasinghe and Maharachchikumbura 2023, Dissanayake et al. 2024; Wanasinghe et al. 2024). Notably, our results underscore the effectiveness of ITS and *rpb*2 loci in distinguishing species within the genera *Parastagonospora* and *Phaeoseptoriella*, supporting prior research on their importance for precise species identification (Quaedvlieg et al. 2013; Crous et al. 2019). However, we found that sequences from LSU and SSU alone often do not provide adequate differentiation. There is a notable lack of comprehensive data regarding the *tef*1-α region among the species studied, suggesting that its phylogenetic utility requires further investigation.

Morphologically, *Parastagonospora* species are primarily identified from their asexual states in natural settings, with sexual morphs either rarely observed or under-documented. The identified sexual morphs resemble didymella-like and phaeosphaeria-like structures, characterized by immersed ascomata with slightly papillate ostioles, bitunicate asci, and fusoid, septate ascospores that range from subhyaline to pale brown (Quaedvlieg et al. 2013). In this study, we have isolated eight collections of *Parastagonospora*, all reported from their asexual morphs. We have comprehensively summarized the asexual characteristics of all known *Parastagonospora* species in Table 4. The most common characteristics of these asexual morphs are globose to subglobose, brown or black, and semi- or fully immersed conidiomata, ampulliform, subcylindrical, lageniform, or doliiform conidiogenous cells proliferating percurrently at the apex and cylindrical or subcylindrical, subhyaline or hyaline, granular to multi-guttulate septate conidia (Quaedvlieg et al. 2013; Li et al. 2016; Thambugala et al. 2017; Goonasekara et al. 2019; Brahmanage et al. 2020; Croll et al. 2021). Our two new species fit well within the morphological features representing the genus.

*Loliumperenne* is an important pasture and forage plant used in many pasture seed mixes (Wei et al. 2023) and has been reported to have the potential for phytoremediation of contaminated soils (Yarahmadi et al. 2017; Madni et al. 2021). *Parastagonosporanodorum* was reported on *Loliumperenne* in Denmark by Quaedvlieg et al. (2013). In this study, *Parastagonosporayunnanensis*, is reported on the host plant *Loliumperenne* in China. *Dactylisglomerata* (Poaceae) is considered an economically important grass in grasslands (Nösberger and Opitz von Boberfeld 1986). At present, eight species from the genus *Parastagonospora* have been reported on *Dactylisglomerata* in Italy (Li et al. 2015, 2016; Thambugala et al. 2017; Goonasekara et al. 2019; Brahmanage et al. 2020; Croll et al. 2021). This study also reports *Parastagonosporazhaotongensis*, on the host plant *Dactylisglomerata* in China. Our findings, coupled with the host information for this fungal species presented in Table 1, suggest that there may be widespread interactions between *Parastagonospora* and various grass species across diverse geographic regions. The discovery of *Phaeoseptoriellapoaceicola* on *Dactylisglomerata* marks the first report of any *Phaeoseptoriella* species on this host, suggesting a previously unrecognized host-fungus interaction.

## Supplementary Material

XML Treatment for Parastagonospora yunnanensis

XML Treatment for Parastagonospora zhaotongensis

XML Treatment for Phaeoseptoriella poaceicola

## ﻿Additional information

### Author ORCIDs

Ying Gao https://orcid.org/0000-0001-8671-1978

Tingfang Zhong https://orcid.org/0009-0000-2767-1347

Prapassorn Damrongkool Eungwanichayapant https://orcid.org/0000-0001-8005-4137

Ruvishika S. Jayawardena https://orcid.org/0000-0001-7702-4885

Kevin D. Hyde https://orcid.org/0000-0002-2191-0762

Turki Kh. Faraj https://orcid.org/0000-0002-6012-8474

Dhanushka N. Wanasinghe https://orcid.org/0000-0003-1759-3933

Heng Gui https://orcid.org/0000-0002-0946-1589

### Data availability

All of the data that support the findings of this study are available in the main text.
